# Antioxidant Defense and Transcriptional Reprogramming Account for the Differential Cold Tolerance of Two Japonica Rice Cultivars During Germination Under Low-Temperature Stress

**DOI:** 10.3390/genes17010083

**Published:** 2026-01-13

**Authors:** Ziting Gao, Yulu Shi, Yu Wang, Qingrui Zhang, Qingwang Su, Xiao Han, Fenglou Ling

**Affiliations:** Faculty of Agronomy, Jilin Agricultural University, Changchun 130118, China; gaoziting1119@outlook.com (Z.G.); yulushi85@163.com (Y.S.); 17543559353@163.com (Y.W.); 13171128999@163.com (Q.Z.); sqw19951128@163.com (Q.S.)

**Keywords:** rice, germination stage, low-temperature stress, physiological characteristics, transcriptome analysis

## Abstract

Background: Low-temperature stress represents a significant constraint on rice production, especially during the germination stage. Consequently, comprehending the mechanisms underlying cold tolerance is of utmost importance for the breeding of resilient rice varieties. This research systematically examined the phenotypic and physiological responses of a cold-tolerant cultivar (JND815) and a cold-sensitive cultivar (Jiyu Japonica) to low-temperature stress (15 °C) during the germination process. Methods: Following a 17-day incubation period, physiological analyses were conducted. Transcriptomic analysis was performed to identify differentially expressed genes (DEGs), which were further subjected to KEGG enrichment analysis and Gene Ontology (GO) annotation. Additionally, the expression trends of selected cold-responsive DEGs were verified via qRT-PCR. Results: Following a 17-day incubation period, physiological analyses indicated that, in comparison to the control group (28 °C), the stress treatment notably reduced the activities of superoxide dismutase (SOD) and catalase (CAT), while increasing the activity of peroxidase (POD) and the content of malondialdehyde (MDA). Significantly, JND815 accumulated a substantially lower amount of MDA than Jiyu Japonica, suggesting superior membrane stability and oxidative stress tolerance. Transcriptomic analysis identified 11,234 and 14,164 differentially expressed genes (DEGs) in JND815 and Jiyu Japonica, respectively. KEGG enrichment analysis demonstrated that these DEGs were significantly associated with phenylpropanoid biosynthesis and carbon metabolism, and Gene Ontology (GO) annotation classified them into biological processes, cellular components, and molecular functions. The expression trends of six cold-responsive DEGs were verified by qRT-PCR to be consistent with the transcriptomic data. Conclusions: These findings offer insights into the molecular mechanisms of the low-temperature response during rice germination and lay a foundation for the genetic improvement of cold-tolerant rice varieties.

## 1. Introduction

Rice (*Oryza sativa* L.), a staple food crop, holds significant importance for global food security [1]. Nevertheless, its cultivation and productivity are often restricted by low-temperature stress, especially during the sensitive early growth phases such as germination and seedling establishment [2]. Exposure to chilling stress may result in delayed germination, poor seedling vitality, and even seedling death, causing irreversible harm to crop stand establishment and subsequent yield [3]. In China, annual yield losses due to low-temperature injury are estimated to be 3–5 billion kilograms, with reduction rates reaching approximately 40% in severe instances [4,5]. These considerable losses not only endanger rice productivity but also impede the potential for expanding cultivation into cooler regions [6,7]. Therefore, clarifying the molecular mechanisms underlying cold tolerance during germination and developing resilient varieties are of crucial significance for stabilizing rice production. This is particularly urgent in the context of promoting water-efficient direct-seeding cultivation and adapting to the unpredictable challenges presented by climate change [8,9,10].

At the physiological level, low-temperature stress perturbs crucial metabolic processes in germinating seeds, encompassing the metabolism of starch and soluble sugars. This perturbation impairs the energy supply by disrupting respiratory metabolism and ATP synthesis [11,12,13]. A characteristic feature of cold-induced damage is membrane lipid peroxidation, in which the accumulation of malondialdehyde (MDA) acts as a pivotal biomarker for membrane system impairment [14,15]. Genetically, cold tolerance is acknowledged as a complex quantitative trait regulated by multi-gene networks and substantially influenced by genotype-environment interactions [16]. Although considerable knowledge has been acquired through quantitative trait locus (QTL) mapping, functional investigations of key transcription factors (e.g., the bZIP factor CHS1 as a negative regulator) [17], and the dissection of conserved signaling pathways such as the ICE-CBF-COR regulatory module [18], the preponderance of this research has focused on the seedling and adult stages, with the germination stage remaining relatively under-investigated.

Although transcriptomic investigations have offered valuable perspectives on the low-temperature responses of rice [19], a considerable number of studies have concentrated on individual cultivars or isolated physiological processes. These studies lack systematic comparisons between genetically distinct cultivars adapted to specific agro-ecological zones. For example, the previous research conducted by Pan et al. failed to integrate physiological indicators with genome-wide transcriptomic data to identify practical markers for breeding. Furthermore, there is a significant lacuna in research regarding the post-germination seedling stage, which is a crucial period for successful field establishment and final yield, particularly in high-latitude cold regions such as Northeast China. This study examines the japonica rice cultivar pair JND815 and Jiyu Japonica via transcriptomic analysis coupled with physiological assays, to dissect cold tolerance mechanisms during the dark germination stage critical to direct-seeding systems in cold regions. Consequently, the molecular mechanisms accounting for genotypic differences in cold tolerance during this critical stage, as well as integrated physiological-molecular screening frameworks for regionally adapted japonica rice, are still inadequately developed.

The capacity to adapt during the post-germination phase is of great significance as it dictates seedling vigor and membrane stability, and exhibits a strong correlation with subsequent growth and the formation of final yield [20,21]. Transcriptomics has evolved into a potent instrument for elucidating intricate abiotic stress responses [22]. Meanwhile, physiological traits, including the activities of antioxidant enzymes (such as SOD, POD, and CAT) and the content of MDA, offer reliable and quantifiable indicators of oxidative stress and cellular integrity [23]. Nevertheless, only a limited number of studies have synergistically integrated these approaches to establish a robust screening system for locally adapted japonica rice germplasm. This integration is a prerequisite for the efficient breeding of cold-tolerant rice varieties tailored to the distinctive climate of Northeast China.

Drawing upon previous screening experiments, two temperate japonica rice cultivars exhibiting significantly contrasting cold tolerance were selected: the cold-tolerant JND815 and the cold-sensitive Jiyu Japonica. On this basis, the present study is not aimed at exploring cold tolerance mechanisms from scratch. Instead, it is designed to dissect the cultivar-specific physiological and transcriptional responses that underlie their differential tolerance. This targeted comparative approach is expected to generate practical insights for the genetic improvement of cold-tolerant japonica rice.

We posited the following hypotheses: (1) The differential cold tolerance exhibited in seedlings 17 days post-germination is regulated by distinct oxidative stress management mechanisms. Specifically, JND815 sustains more stable activities of crucial antioxidant enzymes (SOD, CAT, POD) and accumulates less MDA under low-temperature conditions, rendering these traits reliable physiological indicators. (2) Comparative transcriptomic profiling will pinpoint cold-responsive genes and pathways that are closely correlated with the superior physiological performance of JND815, thereby uncovering genotype-specific regulatory networks.

To validate these hypotheses, seedlings of the two cultivars were cultivated under low-temperature stress (15 °C) and optimal control (28 °C) conditions for a duration of 17 days. Coleoptile and newly emerged leaf tissues were collected for comprehensive analyses, encompassing physiological measurements (activities of superoxide dismutase (SOD), catalase (CAT), and peroxidase (POD), as well as malondialdehyde (MDA) content) and ribonucleic acid sequencing (RNA-seq). The ultimate objective of this study is to establish an integrated physiological-molecular screening system to expedite the development of cold-tolerant japonica rice varieties suitable for Northeast China.

## 2. Materials and Methods

### 2.1. Plant Materials and Growth Conditions

Two temperate japonica rice (*O. sativa*) cultivars exhibiting contrasting cold-tolerance capabilities were utilized in this study: the cold-tolerant cultivar ‘JND815’ and the cold-sensitive cultivar ‘Jiyu Japonica’. The seeds were supplied by the Rice Research Institute of Jilin Agricultural University.

The experiment adopted a completely randomized design with two factors: Cultivar (JND815, Jiyu Japonica) and Temperature (Control, Stress). The control group was kept at an optimal temperature of 28 °C, whereas the stress group was exposed to a low temperature of 15 °C. Each treatment combination incorporated three biological replicates. Each biological replicate was derived from an independent seed batch and was processed independently throughout the experiment to guarantee statistical independence.

Plump and uniform seeds were selected from each cultivar. Surface sterilization was performed by immersing the seeds in 75% (*v*/*v*) ethanol for 20 min, followed by 3–5 rinses with sterile distilled water to remove residual ethanol.

For each biological replicate, 50 sterilized seeds were evenly placed on a sterile Petri dish (90 mm diameter) lined with two layers of filter paper. Each dish was supplemented with 50 mL of sterile distilled water and incubated at 28 °C in the dark for 48 h to allow uniform imbibition. After imbibition, the seeds were transferred to new sterile Petri dishes containing a single layer of moistened filter paper. During the subsequent culture period, each dish was supplied with 5–10 mL of sterile distilled water every two days to maintain humidity and minimize microbial contamination.

The Petri dishes were then transferred to plant growth chambers set to the respective treatment temperatures (28 °C or 15 °C). All treatments were conducted in the dark to prevent photosynthesis and associated confounding effects. The 17-day incubation period was selected to cover the critical early seedling development stage [24,25]. This dark incubation regime effectively mimics the subterranean environment of seeds during field emergence under direct-seeding conditions, where germination and early seedling growth occur without light, thereby providing a physiologically relevant context for assessing cold tolerance applicable to agricultural practice. Based on previous studies indicating that 15 °C effectively induces cold stress at germination [26,27]. Germination, defined as radicle protrusion ≥ 1 mm (according to commonly used germination assessment guideline recommended by the International Rice Research Institute (IRRI)), was monitored daily. The final germination rate was calculated on day 7 as (Number of germinated seeds/Total sown seeds) × 100%.

After 17 days of treatment, seedlings were harvested. For each biological replicate, coleoptiles and newly emerged true leaves were pooled from five uniformly developed seedlings (with coleoptiles elongated to 3–4 cm and newly emerged leaves measuring 1–2 cm in length) to ensure tissue comparability. A total of 0.1 g of the pooled fresh tissue per replicate was immediately flash-frozen in liquid nitrogen and stored at −80 °C for subsequent physiological and transcriptomic analyses.

### 2.2. Physiological and Biochemical Analyses

The activities of antioxidant enzymes—superoxide dismutase (SOD), peroxidase (POD), and catalase (CAT)—as well as the content of malondialdehyde (MDA), were measured using commercial assay kits (Beijing Solabao Technology Co., Ltd., Beijing, China; Cat. Nos.: BC0170 for SOD, BC0090 for POD, BC0200 for CAT, and BC0020 for MDA).

Approximately 0.1 g of frozen tissue was ground to a fine powder in liquid nitrogen. The powder was homogenized in 1 mL of ice-cold extraction buffer (50 mM phosphate buffer, pH 7.8, containing 1% polyvinylpyrrolidone and 1 mM EDTA) and centrifuged at 12,000× *g* for 15 min at 4 °C. The resulting supernatant was used for the assays. SOD activity was determined by measuring the inhibition of photochemical reduction in nitroblue tetrazolium (NBT) at 560 nm. POD activity was assayed by monitoring the oxidation of guaiacol at 470 nm. CAT activity was evaluated by tracking the decomposition of H_2_O_2_ at 240 nm. MDA content, an indicator of lipid peroxidation, was measured using the thiobarbituric acid (TBA) reaction method, with absorbance read at 532 nm and 600 nm for correction.

Enzyme activities were expressed in units per gram of fresh weight (U g^−1^ FW), and MDA content was expressed as nanomoles per gram of fresh weight (nmol g^−1^ FW). All measurements were performed with three technical replicates per biological sample. Quality control included generating standard curves with R^2^ ≥ 0.99 for all assays and ensuring a coefficient of variation (CV) of less than 10% among technical replicates.

### 2.3. RNA Extraction, Library Construction, and Transcriptome Sequencing

Total RNA was extracted from the pooled seedling tissues using the Tissue Total RNA Isolation Kit (Sangon Biotech Shanghai Co., Ltd., Shanghai, China, Cat. No. B511311). RNA integrity was verified using an Agilent 2100 Bioanalyzer, ensuring all samples had an RNA Integrity Number (RIN) greater than 7.5.

A total of 12 cDNA libraries (2 cultivars × 2 temperature treatments × 3 biological replicates) were constructed using the Illumina TruSeq(Illumina, Inc., San Diego, CA, USA) RNA Sample Preparation Kit according to the manufacturer’s instructions. Sequencing was performed on an Illumina HiSeq™ platform, generating 150 bp paired-end reads.

### 2.4. Bioinformatic Analysis of RNA-Seq Data

Raw sequencing reads were first processed to ensure data quality. Adapters and low-quality bases (Q-score < 20) were trimmed using Trimmomatic (v0.39). The clean reads were then aligned to the *O. sativa* subsp. *japonica* reference genome (cv. Nipponbare, IRGSP-1.0, GCF_001433935.1) using HISAT2 (v2.1.0) with the --dta-cufflinks parameter [19]. The alignment statistics, including mapping rates, were assessed using RSeQC. Read counts for each gene were generated from the alignment files using HTSeq-count (v0.13.5) [28], while FPKM (Fragments Per Kilobase of transcript per Million mapped reads) for expression profiling was calculated using Cufflinks (v2.2.1) [29]. Differential expression analysis was performed using the DESeq2 (v1.30.1) package in R [30], which employs a negative binomial generalized linear model. A factorial design formula (~Cultivar + Temperature + Cultivar:Temperature) was applied to analyze the main effects of cultivar and temperature, as well as their interaction effect, to test cultivar-specific stress responses (interaction term). Genes with an absolute log2 fold change |≥1| and an adjusted *p*-value (Benjamini–Hochberg FDR) < 0.05 were considered differentially expressed genes (DEGs).

Gene Ontology (GO) enrichment analysis and Kyoto Encyclopedia of Genes and Genomes (KEGG) pathway enrichment analysis were conducted for the DEG sets using the clusterProfiler (v4.0) R package. Terms with an adjusted *p*-value (Q value) < 0.05 were considered significantly enriched.

### 2.5. Quantitative Real-Time PCR (qRT-PCR) Validation

To validate the RNA-seq results, six DEGs (*Os01g18900*, *Os03g25370*, *Os03g25360*, *Os01g70520*, *Os04g43360*, *Os05g30350*) were selected for qRT-PCR analysis. Gene-specific primers were designed using Primer Premier 5.0 software (Supplementary Table S1). First-strand cDNA was synthesized from 1 μg of total RNA using the TaKaRa PrimeScript RT reagent kit (TaKaRa Bio Inc., Shiga, Japan, Cat. No. Trans AT311-03). qRT-PCR was performed using the Premix Ex Taq™ II kit (TaKaRa Bio Inc., Cat. No. RR820B) on a QuantStudio 5 Real-Time PCR System (Applied Biosystems, Waltham, MA, USA). The 20 μL reaction mixture contained 1 μL of cDNA, 10 μL of Premix, 0.8 μL of each primer (10 μmol/L), 0.4 μL of Rox reference dye, and 7 μL of ddH_2_O. The thermal cycling conditions were initial denaturation at 95 °C for 1 min; 40 cycles of 95 °C for 15 s, 55 °C for 30 s, and 72 °C for 60 s; followed by a melt curve analysis stage. The *Actin1* gene (LOC_Os03g50885) was used as the internal reference details are in Supplementary Table S6 [31,32,33].

## 3. Results

### 3.1. Phenotypic Responses to Low-Temperature Stress

**Figure 1:**
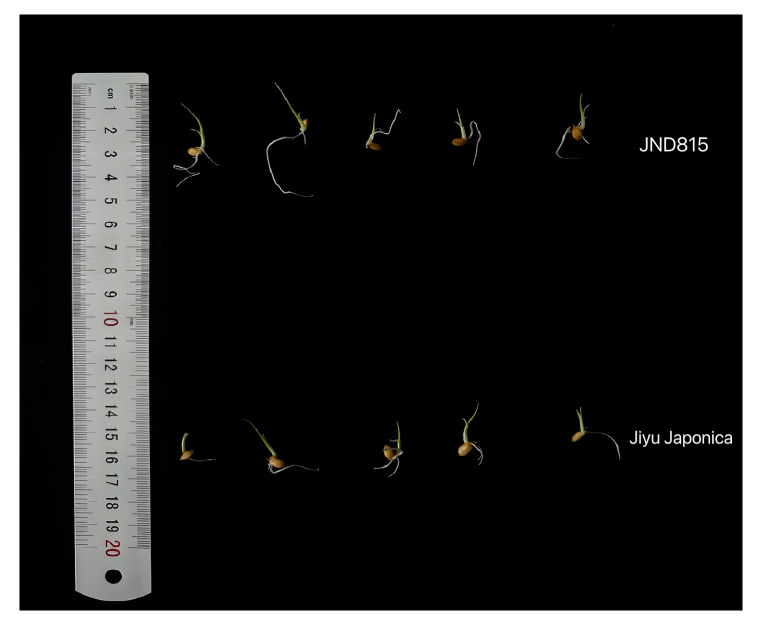
Phenotypic performance of rice seedlings at 17 days after germination under low temperature stress in JND815 and Jiyu Japonica.

Under optimal temperature conditions (28 °C), no significant disparities in germination or seedling growth were detected between JND815 and Jiyu Japonica. Conversely, exposure to low-temperature stress (15 °C) for 17 days notably inhibited the growth of both cultivars. Phenotypic evaluation indicated that JND815 sustained significantly superior seedling growth vigor compared to Jiyu Japonica under stress conditions (Figure 1). Quantitative analyses further corroborated that JND815 manifested significant advantages in crucial agronomic traits, encompassing final germination rate, plant fresh and dry weights, and root and shoot lengths (Table 1), collectively suggesting its stronger cold tolerance during the germination stage.

### 3.2. Comparative Analysis of Physiological Indicators

**Figure 2:**
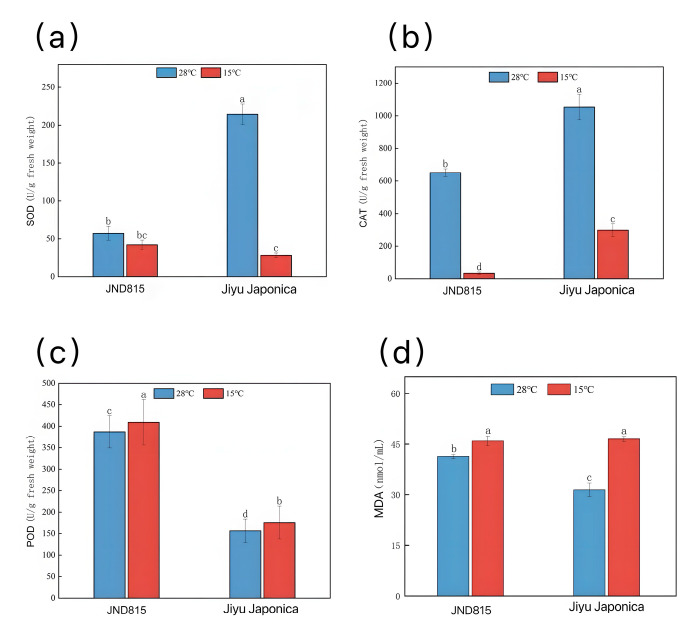
Effects of cold stress on physiological indicators of JND815 and Jiyu Japonica (**a**) SOD activity; (**b**) CAT activity; (**c**) POD activity; (**d**) MDA content. Notes: (1) Error bars represent the standard deviation (SD) of 3 biological replicates (sample size: *n* = 3); (2) Statistical analysis was performed using independent samples *t*-test (for pairwise comparisons among groups); (3) Different lowercase letters (a, b, c, d) in the figure indicate significant differences among groups at *p* < 0.05.

Analysis of the key physiological indicators associated with oxidative stress demonstrated distinct responses between the two cultivars under low-temperature stress (Figure 2). The activity of superoxide dismutase (SOD) decreased marginally in JND815 but declined significantly in Jiyu Japonica (Figure 2a). The activity of catalase (CAT) was significantly downregulated in both cultivars (Figure 2b), while the activity of peroxidase (POD) increased substantially in both (Figure 2c). Overall, JND815 maintained higher activities of SOD and POD under cold stress compared to Jiyu Japonica. The content of malondialdehyde (MDA), an indicator of membrane lipid peroxidation, increased in both cultivars under cold stress; however, the accumulation was significantly greater in Jiyu Japonica than in JND815 (Figure 2d).

Noteworthily, this physiological advantage might be associated with the phenylpropanoid pathway, which is a conserved element of cold-stress acclimation networks [34]. Our transcriptomic data demonstrate that the key biosynthetic genes in this pathway were markedly upregulated in JND815, whereas their expression was relatively weak in Jiyu Japonica, with detailed information on these differentially expressed genes provided in Supplementary Table S4, suggesting robust activation of the pathway in the cold-tolerant cultivar. Previous studies have indicated that the major metabolites of this pathway (lignins and flavonoids) play crucial roles in plant stress responses: lignins strengthen the associations between cell walls and membranes, alleviating cold-induced structural damage, and flavonoids are effective scavengers of reactive oxygen species (ROS). The well-established functional role of phenylpropanoid metabolites offers a reasonable explanation for why JND815, with more robust pathway activation, accumulated less MDA under low temperatures.

### 3.3. Transcriptomic Analysis of Low-Temperature Stress Responses

#### 3.3.1. RNA-Seq Data Quality and Alignment

Transcriptome sequencing of 12 complementary deoxyribonucleic acid (cDNA) libraries (three biological replicates for each cultivar under each treatment) generated approximately 116.5 gigabytes (Gb) of raw data. Following rigorous quality control, 107.7 Gb of high-quality clean data were acquired, with Q30 scores surpassing 93.51% and guanine-cytosine (GC) contents ranging from 52.58% to 56.71% (Table 2). The clean reads were effectively mapped to the reference genome (Nipponbare), and the mapping rate exceeded 96.14% for all samples (Table 3).

#### 3.3.2. Identification of Differentially Expressed Genes (DEGs)

**Figure 3:**
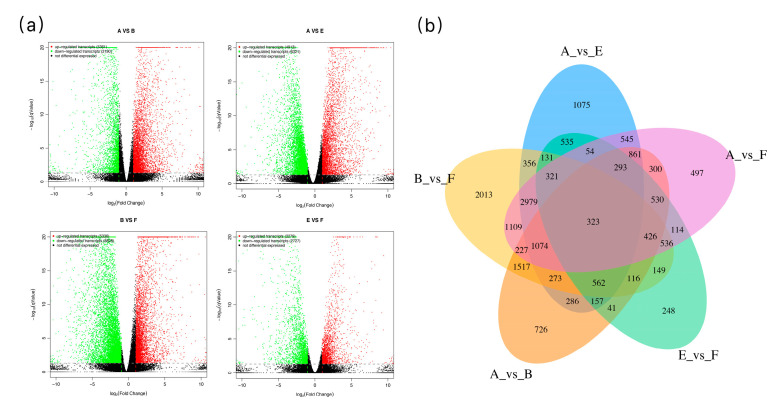
(**a**), Volcano plot of gene expression differences between different treatments; (**b**), Wayne plots of DEGs under different treatments.

Differential expression analysis discerned 14,164 differentially expressed genes (DEGs) (5336 up-regulated; 8828 down-regulated) in Jiyu Japonica and 11,234 DEGs (4913 up-regulated; 6321 down-regulated) in JND815 under low-temperature stress (Table 4). A cumulative total of 8551 DEGs were shared by both cultivars (Figure 3a). The quantity of DEGs between the two cultivars under control conditions was 248, which escalated to 726 under cold stress (Figure 3b).

**Table 4:** Differentially expressed gene statistics.

Compare Type	Up-Regulated	Down-Regulated	Number	Biological Meaning (Sample Combination)
A vs. B	5361	3190	8551	DEGs distinguishing cold-tolerant (JND815) and cold-sensitive (Jiyu Japonica) cultivars under low-temperature stress (A: JND815—15 °C; B: Jiyu Japonica—15 °C)
A vs. E	4913	6321	11,234	Stress-responsive DEGs in cold-tolerant cultivar JND815 (A: JND815—15 °C stress; E: JND815—28 °C control)
B vs. F	5336	8828	14,164	Stress-responsive DEGs in cold-sensitive cultivar Jiyu Japonica (B: Jiyu Japonica-15 °C stress; F: Jiyu Japonica—28 °C control)
E vs. F	2279	2727	5006	Inherent DEGs between two cultivars under normal control conditions (E: JND815—28 °C; F: Jiyu Japonica—28 °C)

#### 3.3.3. Functional Enrichment Analysis of DEGs

**Figure 4:**
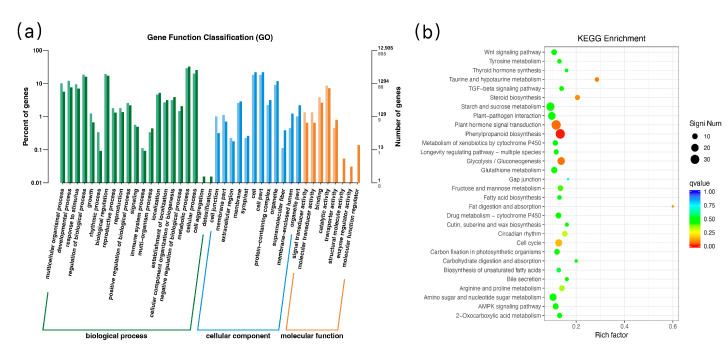
(**a**), Annotation of GO function of DEGs of JND815 and Jiyu Japonica under low temperature stress; (**b**), analysis of KEGG enrichment of DEGs in JND815 and Jiyu Japonica under low temperature stress.

Gene Ontology (GO) enrichment analysis classified the differentially expressed genes (DEGs) of JND815 and Jiyu Japonica under low-temperature stress into three functional domains: biological process, cellular component, and molecular function (Figure 4a). A total of 3246 DEGs were successfully annotated, with the DEGs primarily enriched in metabolic processes (439), cellular processes (306), and biological regulation (257) (complete gene lists are provided in Supplementary Table S2). Kyoto Encyclopedia of Genes and Genomes (KEGG) pathway analysis identified 253 enriched pathways, among which 12 were significantly enriched (Q value ≤ 0.05, Table 5), including phenylpropanoid biosynthesis, plant hormone signal transduction, starch and sucrose metabolism, and carbon metabolism (Figure 4b; Supplementary Table S3). The phenylpropanoid biosynthesis pathway exhibited a high level of enrichment, with key genes demonstrating specific up-regulation in JND815. Notably, starch and sucrose metabolism (68 DEGs) and glutathione metabolism (42 DEGs) contained the most enriched DEGs, highlighting their critical roles in cold stress response.

Both cultivars activated fundamental biological pathways (e.g., metabolic processes, cellular processes) under low-temperature stress. Nevertheless, in comparison to Jiyu Japonica, JND815 had a significantly greater number of genes annotated to signal transducer activity (16 genes) and molecular sensor function (16 genes). An analysis of the key genes driving the enrichment of the “signal transducer activity” (GO:0004871) term indicated that receptor-like kinase genes (e.g., *OsRLK1* and *OsRLK2*), calcium signal-sensing protein genes (e.g., *OsCML1*), and histidine kinase genes were consistently up-regulated in JND815. Additionally, highly expressed *OsRLK1* in JND815 showed a co-expression trend with downstream specific MAPK cascade genes (e.g., *OsMPK3*). This implies that JND815 may possess an enhanced ability for extracellular signal perception and transduction, which may be a key mechanism enabling it to quickly initiate cold stress response and maintain growth.

### 3.4. Validation of RNA-Seq Data by qRT-PCR

**Figure 5:**
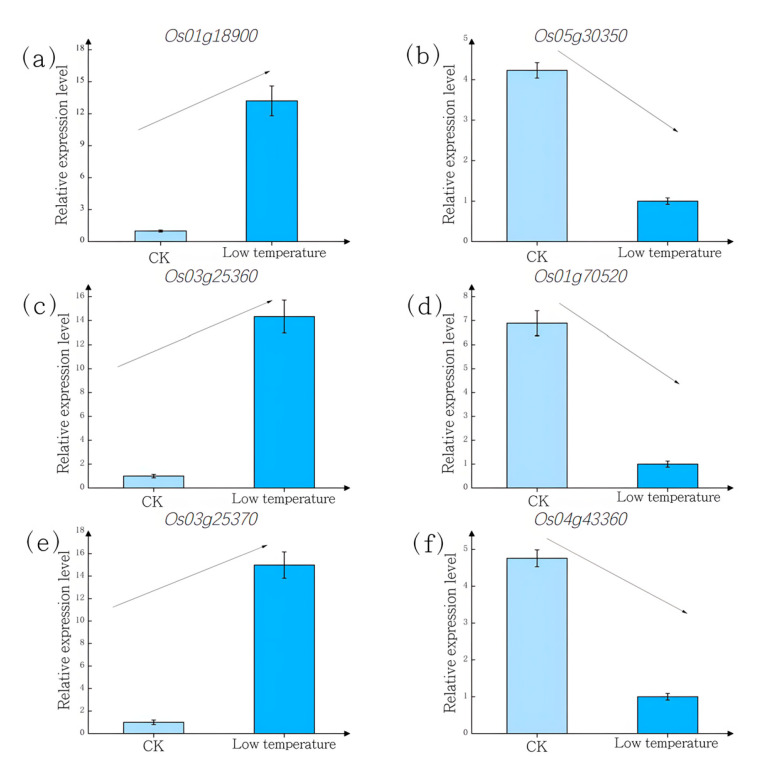
RT-qPCR Verification of DEGs (CK: 28 °C control; Low temperature: 15 °C stress): (**a**) *Os01g18900*; (**b**) *Os03g25370*; (**c**) *Os03g25360*; (**d**) *Os01g70520;* (**e**) *Os04g43360*; (**f**) *Os05g30350*.

To validate the reliability of the transcriptomic sequencing data and to probe the key mechanistic insights, six DEGs were strategically selected for qRT-PCR analysis based on a multi-tiered rationale. The selection criteria included (i) significant differential expression (absolute log2FoldChange > 1 and FDR < 0.05) in both cultivars under cold stress; (ii) functional annotation as key enzymes or regulators within the significantly enriched KEGG pathways identified in our analysis, specifically targeting phenylpropanoid biosynthesis (e.g., *Os03g25370*) and plant hormone signal transduction (e.g., *Os04g43360*) to address the key mechanistic claims; and (iii) exhibiting notable expression divergence between JND815 and Jiyu Japonica, suggesting their potential role in cultivar-specific cold tolerance. The expression trends of six selected DEGs (*Os01g18900*, *Os03g25370*, *Os03g25360*, *Os01g70520*, *Os04g43360*, *Os05g30350*) were highly consistent between the qRT-PCR and RNA-seq data (Figure 5), confirming the reliability of the transcriptomic analyses.

## 4. Discussion

Elucidating the molecular mechanisms of cold tolerance during the germination phase of rice is of paramount importance for the breeding of resilient rice varieties. This significance is particularly pronounced in the context of the growing prevalence of direct-seeding practices and the challenges presented by climate change. Although the adverse effects of low-temperature stress on rice have been extensively documented, the specific mechanisms accounting for cultivar differences at the critical germination stage remain relatively underexplored. Our integrated physiological and transcriptomic analysis reveals that the superior cold tolerance of the japonica cultivar JND815, when compared to Jiyu Japonica, is not simply a result of developmental delay. Instead, it is associated with more efficient oxidative stress management and distinct transcriptional reprogramming [35,36].

A crucial query pertains to whether the observed phenotypic benefits signify a bona fide stress adaptation or merely a passive deceleration of development. Our physiological data refute the latter hypothesis. The notably lower accumulation of malondialdehyde (MDA) in JND815 under cold stress implies active membrane protection that transcends a general growth retardation. This discovery is supported by the cultivar’s consistently higher activities of superoxide dismutase (SOD) and peroxidase (POD), suggesting a proactive mechanism for the scavenging of reactive oxygen species (ROS). These physiological indicators (MDA content, SOD/POD activity) are acknowledged as reliable biomarkers for cold tolerance screening, as their levels are strongly correlated with germination success under stress [37,38,39]. Consequently, the transcriptomic disparities we identified are more likely to mirror a divergent, genetically programmed stress response strategy between the two cultivars. The observation that JND815 demonstrated a more targeted response (11,234 differentially expressed genes, DEGs) compared to the broader reaction in Jiyu Japonica (14,164 DEGs), with stronger enrichment in signal transducer activity, suggests a more precise and potentially efficacious adaptation mechanism.

Our research expands upon prior investigations in several crucial dimensions. Firstly, in contrast to the study conducted by Pan et al. [19], which centered on a single cultivar, our comprehensively characterized cultivar pair presents a distinct comparative framework, unveiling notable phenotypic divergence at the germination stage. Secondly, our dual comparative framework (within and between cultivars under stress conditions) has revealed cultivar-specific pathway activation. For example, the phenylpropanoid biosynthesis pathway was significantly more highly enriched in JND815, with 27 pathway genes specifically up-regulated, a discovery that has not been previously reported. Thirdly, we have identified novel family-level reprogramming within the phenylpropanoid pathway in JND815, providing more profound insights into the regulatory networks underlying cold tolerance.

The comparative transcriptomics approach has been demonstrated to be effective in clarifying the molecular responses to environmental stresses among diverse species [40,41,42,43,44,45]. Our findings are consistent with and expand upon the existing knowledge that rice’s responses to low temperature entail the regulation of multiple pathways, such as hoaling, photosynthesis, and secondary metabolism [46,47]. Nevertheless, while previous studies predominantly concentrated on the seedling or adult stages, our research targeted the relatively under-explored germination stage. We discovered that an elevated capacity for signal perception and transduction, as indicated by the enrichment of receptor-like kinases and calcium signaling genes in JND815, might be a crucial early factor distinguishing the responses of different cultivars. This enhanced signaling could trigger the more efficient downstream physiological responses that we observed. The synergistic effect of key pathways—phenylpropanoid biosynthesis strengthening cell walls and scavenging reactive oxygen species (ROS), hormone signaling (e.g., abscisic acid and jasmonic acid pathways) activating cold-responsive genes to maintain antioxidant activity [48,49], and starch/sucrose metabolism supplying energy and osmolytes—establishes a coherent “molecular regulation → physiological performance” axis that supports JND815’s cold tolerance.

The findings of this study exhibit both consistencies with and substantial expansions upon previous research. For instance, whereas Li et al. [50] reported the up-regulation of abscisic acid (ABA) biosynthesis genes in cold-tolerant rice, our study analogously detected significant enrichment of hormone signaling pathways, corroborating the conserved role of hormonal regulation. Moreover, the quantitative real-time polymerase chain reaction (qRT-PCR) validation of six strategically chosen differentially expressed genes (DEGs) not only verified the technical reliability of our transcriptomic data but also furnished supportive evidence for our key mechanistic assertions. The confirmed up-regulation of genes associated with phenylpropanoid biosynthesis and hormone signaling reinforces the linkage between the transcriptomic profiles and the observed physiological disparities.

Notwithstanding these insights, the present study exhibits certain limitations. The transcriptomic profiles were derived from a mixture of coleoptiles and emerging leaves. Although sampling at a standardized time point mitigates the confounding effects of developmental stages, the specific contributions of distinct cell types still await dissection. Moreover, the cold tolerance of JND815 is presumably polygenic, potentially encompassing known quantitative trait loci (QTLs) (e.g., qLTG3, qSCT1). Consequently, the findings of this study and the proposed physiological-transcriptomic screening model are most directly applicable to the japonica subpopulation. Their generalizability to indica rice or other genetic backgrounds necessitates further validation.

In light of these considerations, this paper presents a well-defined research trajectory for the future. It is imperative to prioritize the functional validation of key candidate genes (such as the identified receptor-like kinases (RLKs) and calcium sensors) through genetic methodologies. Subsequently, conducting association analyses across diverse germplasm resources can help identify deployable haplotype markers associated with superior germination performance under cold stress. Finally, phenotypic correlation studies under field-simulated conditions are indispensable for bridging the gap between molecular markers and the agronomically crucial trait of seedling establishment.

In summary, this study offers an integrated physiological and transcriptomic framework for elucidating the cold-tolerance mechanism in japonica rice during the germination stage. The identified key pathways and candidate genes not only enhance our understanding of cold adaptation but also provide valuable resources and practical biomarkers for expediting the breeding of cold-tolerant rice varieties, which may have potential implications for stabilizing rice production in cold regions.

## 5. Conclusions

This research systematically explored the divergence in cold tolerance during the germination stage between two pre-screened temperate japonica rice cultivars, namely the cold-tolerant JND815 and the cold-sensitive Jiyu Japonica, through the integration of phenotypic, physiological, and transcriptomic analyses. The results of this study offer clear evidence that the enhanced cold tolerance of JND815 is supported by a combination of improved oxidative stress management and distinct transcriptional reprogramming, rather than simply a developmental delay.

At the physiological level, JND815 mitigated the accumulation of reactive oxygen species (ROS) under low-temperature stress by sustaining higher activities of key antioxidant enzymes, namely superoxide dismutase (SOD) and peroxidase (POD). This robust antioxidant defense mechanism was correlated with significantly lower malondialdehyde (MDA) content and enhanced membrane stability in comparison to Jiyu Japonica, validating its superior ability to alleviate cold-induced oxidative damage.

At the molecular level, transcriptome analysis identified 11,234 and 14,164 differentially expressed genes (DEGs) in JND815 and Jiyu Japonica, respectively, under low-temperature stress. Gene Ontology (GO) annotation demonstrated that these DEGs were mainly involved in fundamental biological processes, cellular components, and molecular functions. Kyoto Encyclopedia of Genes and Genomes (KEGG) pathway enrichment analysis revealed the significant activation of key pathways, including phenylpropanoid biosynthesis and carbon metabolism, in both cultivars. Notably, the phenylpropanoid pathway was strongly and specifically up-regulated in JND815, which is consistent with its observed physiological advantages and implies a crucial role in cold adaptation. The reliability of the transcriptomic data was verified by qRT-PCR validation of six selected cold-responsive DEGs.

In conclusion, this study employs an integrated physiology-transcriptome approach to unveil the physiological and transcriptional characteristics related to cold tolerance in JND815 during the crucial germination stage. The identified cold-responsive pathways and candidate genes furnish a theoretical foundation and valuable genetic resources for subsequent research on rice cold tolerance, such as gene cloning and functional verification.

Prospectively, the physiological indices (e.g., malondialdehyde (MDA) content, peroxidase (POD) activity) and molecular signatures (e.g., genes in the phenylpropanoid pathway) identified herein present practical, high-throughput tools for screening cold-tolerant rice germplasm in breeding programs. These physiological indices can be directly incorporated into existing cold tolerance screening workflows for japonica rice to facilitate efficient selection of cold-resilient varieties. Future research ought to prioritize the functional validation of key candidate genes via genetic methods and assess the applicability of this screening paradigm in a broader range of germplasm, including indica varieties, to differentiate japonica-specific mechanisms from universal indicators of cold tolerance. This will be indispensable for developing broad-spectrum, cold-resilient rice varieties capable of addressing the challenges of direct-seeding practices and climate change.

## Figures and Tables

**Table 1:** Determination of germination-related and growth traits of two japonica rice cultivars under 15 °C low-temperature stress.

Measured Parameters	Unit	JND815 (Low-Temperature, 15 °C)	Jiyu Japonica (Low-Temperature, 15 °C)
Germination rate	%	89.2 ± 3.1 a	43.0 ± 9.0 b
Mean germination days	d	5.2 ± 0.3 a	7.8 ± 0.7 b
Root length	cm	3.56 ± 0.19 a	3.26 ± 0.32 b
Shoot length	cm	2.15 ± 0.21 a	1.95 ± 0.59 b
Total fresh weight	mg	18.64 ± 1.23 a	9.18 ± 1.84 b
Root fresh weight	mg	8.21 ± 0.92 a	2.86 ± 0.93 b
Shoot fresh weight	mg	10.43 ± 0.81 a	6.32 ± 0.91 b
Total dry weight	mg	3.52 ± 0.37 a	2.06 ± 0.41 b

Notes: Test materials included cold-tolerant cultivar JND815 and cold-sensitive cultivar Jiyu Japonica, with all seeds cultured in the dark for 17 days under 15 °C low-temperature stress. Measured parameters included germination rate, mean germination days, root length, shoot length, total fresh weight, root fresh weight, shoot fresh weight, and total dry weight. Data are presented as mean ± standard deviation (*n* = 3 biological replicates). Statistical analysis was performed using SPSS 26.0 software with independent samples *t*-test; different lowercase letters (a, b) in the same row indicate significant differences between the two cultivars at *p* < 0.05.

**Table 2:** Sequencing data quality assessment table.

	Total Reads Count	Total Bases Count (bp)	Average Read Length (bp)	Q20 Bases Ratio	Q30 Bases Ratio	GC Bases Ratio
A1	72,687,338	10,520,957,289	144.74	98.24%	94.20%	56.16%
A2	59,412,424	8608,464,948	144.89	98.02%	93.60%	56.71%
A3	67,700,974	9695,924,802	143.22	98.13%	93.87%	55.86%
E1	53,227,322	7652,257,856	143.77	98.35%	94.38%	52.58%
E2	49,067,214	7084,896,887	144.39	98.39%	94.52%	53.44%
E3	39,239,884	5618,508,467	143.18	98.41%	94.56%	54.17%
B1	48,294,724	7008,350,540	145.12	98.00%	93.51%	56.08%
B2	56,732,812	8231,123,682	145.09	98.09%	93.76%	56.42%
B3	44,904,690	6509,328,568	144.96	98.02%	93.55%	56.06%
F1	39,552,138	5653,384,029	142.93	98.34%	94.35%	52.74%
F2	52,834,276	7644,432,305	144.69	98.36%	94.39%	52.69%
F3	57,750,844	8293,887,302	143.61	98.36%	94.36%	53.03%

Notes: A: JND815 under 15 °C treatment; E: JND815 under 28 °C treatment; B: Jiyu Japonica under 15 °C treatment; F: Jiyu Japonica under 28 °C treatment; Numbers 1, 2 and 3 indicate three replications of the samples; Same as below. A detailed cultivar × temperature × replicate mapping table is provided in Supplementary Table S5.

**Table 3:** Comparative analysis with reference group genes.

	Total Reads (Clean Data)	Total Mapped	Reads Mapped in Proper Pairs
A1	71,942,504 (100.00%)	70,110,332 (97.45%)	66,541,184 (92.49%)
A2	58,852,370 (100.00%)	56,725,170 (96.39%)	53,946,428 (91.66%)
A3	64,596,208 (100.00%)	62,619,105 (96.94%)	58,902,582 (91.19%)
E1	51,987,152 (100.00%)	49,980,608 (96.14%)	47,130,890 (90.66%)
E2	47,383,588 (100.00%)	45,885,447 (96.84%)	43,060,142 (90.88%)
E3	39,011,274 (100.00%)	37,970,739 (97.33%)	35,994,748 (92.27%)
B1	47,485,618 (100.00%)	46,563,625 (98.06%)	44,332,016 (93.36%)
B2	55,457,880 (100.00%)	54,441,898 (98.17%)	51,714,998 (93.25%)
B3	44,474,816 (100.00%)	43,624,771 (98.09%)	41,427,258 (93.15%)
F1	38,819,390 (100.00%)	37,695,211 (97.10%)	35,341,350 (91.04%)
F2	51,816,628 (100.00%)	50,478,016 (97.42%)	47,627,252 (91.91%)
F3	56,913,976 (100.00%)	55,252,942 (97.08%)	52,122,424 (91.58%)

**Table 5:** Table of pathway significant enrichment data for each group.

id	Description	Significant	Annotated	Qvalue
ko00480	Glutathione metabolism	42/1108	119/6009	0.0002
ko00982	Drug metabolism-cytochrome P450	33/1108	87/6009	0.0003
ko00400	Phenylalanine, tyrosine and tryptophan biosynthesis	23/1108	54/6009	0.0007
ko00980	Metabolism of xenobiotics by cytochrome P450	29/1108	79/6009	0.0019
ko00460	Cyanoamino acid metabolism	22/1108	59/6009	0.0092
ko00073	Cutin, suberine and wax biosynthesis	14/1108	31/6009	0.0098
ko00710	Carbon fixation in photosynthetic organisms	28/1108	83/6009	0.0098
ko01040	Biosynthesis of unsaturated fatty acids	18/1108	47/6009	0.0165
ko00906	Carotenoid biosynthesis	14/1108	33/6009	0.0172
ko00591	Linoleic acid	10/1108	20/6009	0.0181
ko00500	Starch and sucrose metabolism	68/1108	268/6009	0.0311
ko00030	Pentose phosphate pathway	20/1108	60/6009	0.0492

## Data Availability

These sequence data have been deposited in the NCBI Sequence Read Archive (SRA) database under BioProject accession number PRJNA1392095 (https://www.ncbi.nlm.nih.gov/bioproject/PRJNA1392095, accessed on 18 December 2025).

## Supplementary Materials

The following supporting information can be downloaded at: https://www.mdpi.com/article/10.3390/genes17010083/s1, Table S1: Primers and Sequences Used for Quantitative Real-Time PCR Analysis; Table S2: Annotated list of differential gene GO functions; Table S3: Significant enrichment analysis of differential genes Pathway; Table S4: Detailed comparison of phenylpropanoid pathway and key cold-responsive genes between cold-tolerant (JND815) and cold-sensitive (Jiyu Japonica) rice cultivars during germination under low temperature; Table S5: Mapping of Manuscript Labels to Cultivars, Temperature Treatments, and Replicates; Table S6: Information and Validation Basis of Reference Gene.
