# Supplementary material for: Antioxidant Defense and Transcriptional Reprogramming Account for the Differential Cold Tolerance of Two Japonica Rice Cultivars During Germination Under Low-Temperature Stress
Source: Genes (Basel). 2026 Jan 13;17(1):83. doi: 10.3390/genes17010083 (PMC12841373; doi:10.3390/genes17010083)
Supplement: Supplementary file 1 [file genes-17-00083-s001.zip › genes-4092636-supplementary.pdf]

Supplementary Table S1. Primers and Sequences Used for Quantitative Real-Time PCR Analysis

Primer	GenBank Accession Number	Function	Sequence(5′-3′)
Os01g18900-F	NM_001057718.1	qRT-PCR forward primer	GCTGCTGATGCTGTTCTGCT
Os01g18900-R	NM_001057718.1	qRT-PCR reverse primer	TTTAACGAGCAGCAGCAGCT
Os03g25370-F	NM_001061478.1	qRT-PCR forward primer	TGCTGCTGGTGATGGTGATT
Os03g25370-R	NM_001061478.1	qRT-PCR reverse primer	CAGCAGCAGCTTCTTCTTGG
Os03g25360-F	NM_001061477.1	qRT-PCR forward primer	GATGGTGGTGCTGCTGTTGA
Os03g25360-R	NM_001061477.1	qRT-PCR reverse primer	CTTGCTGCTGCTGCTGTTCT
Os01g70520-F	NM_001425712.1	qRT-PCR forward primer	GCTGCTGATGGTGATGGTGA
Os01g70520-R	NM_001425712.1	qRT-PCR reverse primer	CAGCAGCTTCTTCTTCTTGC
Os04g43360-F	NM_001067336.1	qRT-PCR forward primer	TGATGGTGCTGCTGTTGCTA
Os04g43360-R	NM_001067336.1	qRT-PCR reverse primer	CTTCTTCTTGCAGCAGCAGC
Os05g30350-F	NM_001069805.1	qRT-PCR forward primer	GGTGCTGCTGATGCTGTTGT
Os05g30350-R	NM_001069805.1	qRT-PCR reverse primer	CAGCAGCTTCTTCTTCTTGG
Actin1-F	NM_197285.2	qRT-PCR forward primer	AACTGGTATCGTGCTTGACTCTG
Actin1-R	NM_197285.2	qRT-PCR reverse primer	CGCTTGTTGTGAGGGAATACC

Note: The amplification efficiency of each primer in this table ranges from 92% to 108% (mean 99%), consistent with the technical criteria for reliable qRT-PCR quantification.

Supplementary Table S2. Annotated list of differential gene GO functions

Ontology	Term	GO.ID	Significant	Annotated	Qvalue
biological process	response to stimulus	GO:0050896	151	914	0.000128725
biological process	establishment of localization	GO:0051234	80	412	0.00017073
biological process	localization	GO:0051179	95	672	0.205641176470588
biological process	multi-organism process	GO:0051704	10	57	0.869802824858757
biological process	rhythmic process	GO:0048511	3	12	1
biological process	multicellular organismal process	GO:0032501	89	730	1
biological process	positive regulation of biological process	GO:0048518	36	282	1
biological process	negative regulation of biological process	GO:0048519	33	267	1
biological process	immune system process	GO:0002376	2	12	1
biological process	biological regulation	GO:0065007	257	2232	1
biological process	signaling	GO:0023052	8	64	1
biological process	regulation of biological process	GO:0050789	236	2071	1
biological process	growth	GO:0040007	9	87	1
biological process	reproduction	GO:0000003	15	177	1
biological process	cellular component organization or biogenesis	GO:0071840	47	502	1
biological process	reproductive process	GO:0022414	13	171	1
biological process	metabolic process	GO:0008152	439	4210	1
biological process	cellular process	GO:0009987	306	3310	1
biological process	cell aggregation	GO:0098743	0	2	1
biological process	detoxification	GO:0098754	0	2	1
biological process	membrane	GO:0016020	77	373	2.628e-05
biological	organelle part	GO:0044422	40	286	1

Ontology	Term	GO.ID	Significant	Annotated	Qvalue
process					
biological process	membrane part	GO:0044425	14	92	1
biological process	extracellular region	GO:0005576	4	23	1
biological process	symplast	GO:0055044	5	34	1
biological process	cell junction	GO:0030054	4	41	1
biological process	cell	GO:0005623	342	2854	1
biological process	cell part	GO:0044464	342	2854	1
biological process	supramolecular fiber	GO:0099512	4	50	1
biological process	organelle	GO:0043226	175	1529	1
biological process	membrane-enclose d lumen	GO:0031974	6	160	1
biological process	protein-containing complex	GO:0032991	7	411	1
biological process	transporter activity	GO:0005215	24	103	1
biological process	catalytic activity	GO:0003824	184	933	1
biological process	signal transducer activity	GO:0004871	16	85	1
biological process	molecular transducer activity	GO:0060089	16	85	1
biological process	structural molecule activity	GO:0005198	1	7	1
biological process	molecular function regulator	GO:0098772	1	18	1
biological process	binding	GO:0005488	47	342	1
biological process	enzyme regulator activity	GO:0030234	0	4	1

Supplementary Table S3. Significant enrichment analysis of differential genes Pathway

id	Description	Significant	Annotated	Pvalue	Qvalue
ko00940	Phenylpropanoid biosynthesis	108/1108	254/6009	8.15737278245394e-20	1.88048909406044e-17
ko00196	Photosynthesis - antenna proteins	14/1108	15/6009	6.1107616544948e-10	7.04345685439138e-08
ko00195	Photosynthesis	49/1108	122/6009	1.31590546022802e-08	1.01116945891205e-06
ko04075	Plant hormone signal transduction	85/1108	281/6009	6.0542782618597e-07	3.48917615617704e-05
ko00430	Taurine and hypotaurine metabolism	14/1108	21/6009	1.55048694330467e-06	7.14856085439416e-05
ko00941	Flavonoid biosynthesis	20/1108	38/6009	1.99389056000793e-06	7.66073741476731e-05
ko00592	alpha-Linolenic acid metabolism	24/1108	51/6009	2.61361712922653e-06	8.60725039549789e-05
ko00480	Glutathione metabolism	42/1108	119/6009	7.99259547892719e-06	0.000230312948669086
ko00982	Drug metabolism - cytochrome P450	33/1108	87/6009	1.3478370279029e-05	0.000345235449252321
ko00400	Phenylalanine, tyrosine and tryptophan biosynthesis	23/1108	54/6009	3.22894688076393e-05	0.000744357228302422
ko00980	Metabolism of xenobiotics by cytochrome P450	29/1108	79/6009	8.92959752416776e-05	0.00187137019884473
ko00460	Cyanoamino acid metabolism	22/1108	59/6009	0.000481371575948225	0.00924740132742643
ko00073	Cutin, suberine and wax biosynthesis	14/1108	31/6009	0.00055817243868045	0.00979128052858457
ko00710	Carbon fixation in photosynthetic organisms	28/1108	83/6009	0.000594630278676597	0.00979128052858457
ko01040	Biosynthesis of unsaturated fatty acids	18/1108	47/6009	0.00107415644163654	0.0165080884714668
ko00906	Carotenoid biosynthesis	14/1108	33/6009	0.00119462965054915	0.0172120982546226
ko00591	Linoleic acid	10/1108	20/6009	0.001335269797048	0.018106754523440

id	Description	Significant	Annotated	Pvalue	Qvalue
	metabolism			24	5
ko00500	Starch and sucrose metabolism	68/1108	268/6009	0.00243087201378148	0.0311322205273768
ko00030	Pentose phosphate pathway	20/1108	60/6009	0.00405606922868631	0.0492121418882161
ko00520	Amino sugar and nucleotide sugar metabolism	46/1108	173/6009	0.00462575124124873	0.0533178695701828
ko04626	Plant-pathogen interaction	54/1108	212/6009	0.00599414707712619	0.0655466615637503
ko00402	Benzoxazinoid biosynthesis	3/1108	3/6009	0.00625536633188302	0.0655466615637503
ko00071	Fatty acid degradation	15/1108	47/6009	0.0182637468665447	0.180360919674196
ko00100	Steroid biosynthesis	13/1108	39/6009	0.0187773012263547	0.180360919674196
ko04016	MAPK signaling pathway - plant	37/1108	146/6009	0.022228339036518	0.197488140167797
ko01230	Biosynthesis of amino acids	61/1108	260/6009	0.0222737765394729	0.197488140167797
ko00562	Inositol phosphate metabolism	17/1108	57/6009	0.0245486546175105	0.209596700243072
ko00330	Arginine and proline metabolism	20/1108	71/6009	0.0286919511441073	0.236223206787951
ko00410	beta-Alanine metabolism	14/1108	46/6009	0.0332213614266453	0.26408269155845
ko04920	Adipocytokine signaling pathway	13/1108	43/6009	0.0414251638728718	0.318319680286278
ko03320	PPAR signaling pathway	11/1108	35/6009	0.0447931939037662	0.325251601978144
ko00600	Sphingolipid metabolism	10/1108	31/6009	0.0461004647831532	0.325251601978144
ko04975	Fat digestion and absorption	3/1108	5/6009	0.0465599895982411	0.325251601978144
ko00010	Glycolysis / Gluconeogenesis	35/1108	145/6009	0.0495839504947764	0.336188394995543
ko00590	Arachidonic acid metabolism	5/1108	12/6009	0.0534369882988375	0.351960915411892
ko04973	Carbohydrate digestion and absorption	7/1108	20/6009	0.0597378009602488	0.382531532464751

id	Description	Significant	Annotated	Pvalue	Qvalue
ko01200	Carbon metabolism	61/1108	276/6009	0.0656801111271085	0.409216055102042
ko00561	Glycerolipid metabolism	21/1108	83/6009	0.0731935775884987	0.424666016470233
ko02024	Quorum sensing	16/1108	60/6009	0.0733113911957158	0.424666016470233
ko00905	Brassinosteroid biosynthesis	5/1108	13/6009	0.074061651664802	0.424666016470233
ko00051	Fructose and mannose metabolism	19/1108	74/6009	0.0755284992763268	0.424666016470233
ko00965	Betalain biosynthesis	3/1108	6/6009	0.0805461378173043	0.442095342907009
ko00380	Tryptophan metabolism	14/1108	52/6009	0.0842673236119315	0.451763619853439
ko04911	Insulin secretion	2/1108	3/6009	0.0894132787890946	0.451859741814434
ko01212	Fatty acid metabolism	21/1108	85/6009	0.0901546518941855	0.451859741814434
ko04152	AMPK signaling pathway	25/1108	104/6009	0.0901656197136565	0.451859741814434
ko00625	Chloroalkane and chloroalkene degradation	5/1108	14/6009	0.0983725665718815	0.482499262692991
ko04976	Bile secretion	9/1108	31/6009	0.102045681174023	0.490087810901559
ko04727	GABAergic synapse	10/1108	36/6009	0.111720067790347	0.520030284044528
ko04710	Circadian rhythm	15/1108	59/6009	0.113372126961446	0.520030284044528
ko00945	Stilbenoid, diarylheptanoid and gingerol biosynthesis	7/1108	23/6009	0.1150477957167	0.520030284044528
ko00904	Diterpenoid biosynthesis	11/1108	41/6009	0.119749372079507	0.522155945103581
ko04913	Ovarian Steroidogenesis	3/1108	7/6009	0.12214137869111	0.522155945103581
ko00250	Alanine, aspartate and glutamate metabolism	14/1108	55/6009	0.122313241935222	0.522155945103581
ko00061	Fatty acid biosynthesis	12/1108	46/6009	0.12646325593577	0.53005651770208
ko00052	Galactose metabolism	17/1108	71/6009	0.147332723378691	0.606501248494987
ko00650	Butanoate metabolism	8/1108	29/6009	0.150544769731708	0.608851423291673
ko00903	Limonene and	3/1108	8/6009	0.169653649997781	0.67027228168596

id	Description	Significant	Annotated	Pvalue	Qvalue
	pinene degradation				
ko00270	Cysteine and methionine metabolism	27/1108	122/6009	0.171642626750035	0.67027228168596
ko04211	Longevity regulating pathway - mammal	16/1108	68/6009	0.174454429479907	0.67027228168596
ko00220	Arginine biosynthesis	9/1108	35/6009	0.182650434743612	0.690257898340828
ko00053	Ascorbate and aldarate metabolism	12/1108	51/6009	0.218809494476963	0.813570106798895
ko00630	Glyoxylate and dicarboxylate metabolism	17/1108	76/6009	0.225415190981279	0.824827515871345
ko02010	ABC transporters	9/1108	38/6009	0.256923728456548	0.903010705338699
ko04919	Thyroid hormone signaling pathway	9/1108	38/6009	0.256923728456548	0.903010705338699
ko00280	Valine, leucine and isoleucine degradation	12/1108	53/6009	0.262000523110551	0.903010705338699
ko00680	Methane metabolism	14/1108	63/6009	0.262450371665791	0.903010705338699
ko00960	Tropane, piperidine and pyridine alkaloid biosynthesis	8/1108	34/6009	0.281706073954241	0.911171208653728
ko00360	Phenylalanine metabolism	14/1108	64/6009	0.283143192096275	0.911171208653728
ko01220	Degradation of aromatic compounds	4/1108	15/6009	0.293931053685105	0.911171208653728
ko00760	Nicotinate and nicotinamide metabolism	5/1108	20/6009	0.303061360191559	0.911171208653728
ko00401	Novobiocin biosynthesis	2/1108	6/6009	0.30631126527251	0.911171208653728
ko04068	FoxO signaling pathway	12/1108	56/6009	0.331434402354086	0.911171208653728
ko00232	Caffeine metabolism	1/1108	2/6009	0.334805492604414	0.911171208653728
ko00350	Tyrosine metabolism	10/1108	46/6009	0.336613403971176	0.911171208653728
ko00531	Glycosaminogl	4/1108	16/6009	0.339558338853284	0.911171208653728

id	Description	Significant	Annotated	Pvalue	Qvalue
	ycan degradation				
ko00564	Glycerophospholipid metabolism	24/1108	119/6009	0.347361550483414	0.911171208653728
ko00062	Fatty acid elongation	14/1108	67/6009	0.347901645538581	0.911171208653728
ko04070	Phosphatidylinositol signaling system	14/1108	68/6009	0.370130003389014	0.911171208653728
ko04213	Longevity regulating pathway - multiple species	14/1108	68/6009	0.370130003389014	0.911171208653728
ko00626	Naphthalene degradation	2/1108	7/6009	0.37997921990507	0.911171208653728
ko00740	Riboflavin metabolism	2/1108	7/6009	0.37997921990507	0.911171208653728
ko04668	TNF signaling pathway	2/1108	7/6009	0.37997921990507	0.911171208653728
ko00140	Steroid hormone biosynthesis	4/1108	17/6009	0.385370103283526	0.911171208653728
ko04612	Antigen processing and presentation	12/1108	59/6009	0.404126577238829	0.911171208653728
ko00261	Monobactam biosynthesis	3/1108	13/6009	0.439916146170938	0.911171208653728
ko04925	Aldosterone synthesis and secretion	3/1108	13/6009	0.439916146170938	0.911171208653728
ko00072	Synthesis and degradation of ketone bodies	2/1108	8/6009	0.4500877432075	0.911171208653728
ko00902	Monoterpenoid biosynthesis	2/1108	8/6009	0.4500877432075	0.911171208653728
ko00944	Flavone and flavonol biosynthesis	1/1108	3/6009	0.457501599512073	0.911171208653728
ko04711	Circadian rhythm - fly	1/1108	3/6009	0.457501599512073	0.911171208653728
ko04024	cAMP signaling pathway	11/1108	56/6009	0.461676846611063	0.911171208653728
ko04146	Peroxisome	19/1108	99/6009	0.463863931665035	0.911171208653728
ko00565	Ether lipid metabolism	6/1108	30/6009	0.485848251770413	0.911171208653728
ko00908	Zeatin biosynthesis	6/1108	30/6009	0.485848251770413	0.911171208653728
ko04810	Regulation of actin	10/1108	52/6009	0.497596444695937	0.911171208653728

id	Description	Significant	Annotated	Pvalue	Qvalue
	cytoskeleton				
ko04918	Thyroid hormone synthesis	5/1108	25/6009	0.500394327869383	0.911171208653728
ko00511	Other glycan degradation	4/1108	20/6009	0.518196733992427	0.911171208653728
ko00950	Isoquinoline alkaloid biosynthesis	5/1108	26/6009	0.537794479101736	0.911171208653728
ko00521	Streptomycin biosynthesis	3/1108	15/6009	0.541433268434488	0.911171208653728
ko02020	Two-componen t system	3/1108	15/6009	0.541433268434488	0.911171208653728
ko00040	Pentose and glucuronate interconversion s	16/1108	87/6009	0.54878630517162	0.911171208653728
ko00770	Pantothenate and CoA biosynthesis	6/1108	32/6009	0.553688583534192	0.911171208653728
ko00281	Geraniol degradation	1/1108	4/6009	0.557582889512176	0.911171208653728
ko00901	Indole alkaloid biosynthesis	1/1108	4/6009	0.557582889512176	0.911171208653728
ko04630	Jak-STAT signaling pathway	1/1108	4/6009	0.557582889512176	0.911171208653728
ko04270	Vascular smooth muscle contraction	4/1108	21/6009	0.559485618555979	0.911171208653728
ko04712	Circadian rhythm - plant	7/1108	38/6009	0.567213978463648	0.911171208653728
ko00640	Propanoate metabolism	5/1108	27/6009	0.573846104072192	0.911171208653728
ko00524	Neomycin, kanamycin and gentamicin biosynthesis	2/1108	10/6009	0.575417881518607	0.911171208653728
ko01210	2-Oxocarboxyli c acid metabolism	11/1108	61/6009	0.585120371907197	0.911171208653728
ko00300	Lysine biosynthesis	3/1108	16/6009	0.588019790645535	0.911171208653728
ko00627	Aminobenzoate degradation	3/1108	16/6009	0.588019790645535	0.911171208653728
ko04350	TGF-beta signaling pathway	9/1108	50/6009	0.589727383264347	0.911171208653728
ko04130	SNARE interactions in	7/1108	39/6009	0.596725714775158	0.911171208653728

id	Description	Significant	Annotated	Pvalue	Qvalue
	vesicular transport				
ko00780	Biotin metabolism	4/1108	22/6009	0.598774874441604	0.911171208653728
ko00860	Porphyrin and chlorophyll metabolism	8/1108	45/6009	0.606358725712336	0.911171208653728
ko04912	GnRH signaling pathway	6/1108	34/6009	0.617100620335966	0.911171208653728
ko04910	Insulin signaling pathway	18/1108	102/6009	0.622835018613261	0.911171208653728
ko00910	Nitrogen metabolism	7/1108	40/6009	0.625171037956362	0.911171208653728
ko00603	Glycosphingolipid biosynthesis - globo series	2/1108	11/6009	0.629769304321905	0.911171208653728
ko04750	Inflammatory mediator regulation of TRP channels	3/1108	17/6009	0.63144472365168	0.911171208653728
ko04922	Glucagon signaling pathway	15/1108	86/6009	0.638670459537025	0.911171208653728
ko00930	Caprolactam degradation	1/1108	5/6009	0.63921455619336	0.911171208653728
ko04310	Wnt signaling pathway	16/1108	92/6009	0.645259316543606	0.911171208653728
ko04728	Dopaminergic synapse	8/1108	47/6009	0.658037599589228	0.911171208653728
ko00643	Styrene degradation	3/1108	18/6009	0.671583798075551	0.911171208653728
ko00983	Drug metabolism - other enzymes	3/1108	18/6009	0.671583798075551	0.911171208653728
ko04540	Gap junction	3/1108	18/6009	0.671583798075551	0.911171208653728
ko04745	Phototransduction - fly	2/1108	12/6009	0.678531537797915	0.911171208653728
ko04917	Prolactin signaling pathway	2/1108	12/6009	0.678531537797915	0.911171208653728
ko04970	Salivary secretion	2/1108	12/6009	0.678531537797915	0.911171208653728
ko04261	Adrenergic signaling in cardiomyocytes	7/1108	42/6009	0.67853628483386	0.911171208653728
ko04020	Calcium signaling pathway	8/1108	48/6009	0.68231646319387	0.911171208653728

id	Description	Significant	Annotated	Pvalue	Qvalue
ko00900	Terpenoid backbone biosynthesis	10/1108	60/6009	0.690308405259167	0.911171208653728
ko04391	Hippo signaling pathway -fly	5/1108	31/6009	0.701518719154805	0.911171208653728
ko00310	Lysine degradation	6/1108	37/6009	0.701870260833332	0.911171208653728
ko04210	Apoptosis	4/1108	25/6009	0.703113988172288	0.911171208653728
ko04214	Apoptosis - fly	4/1108	25/6009	0.703113988172288	0.911171208653728
ko04614	Renin-angiotensin system	1/1108	6/6009	0.70579521437753	0.911171208653728
ko04725	Cholinergic synapse	1/1108	6/6009	0.70579521437753	0.911171208653728
ko04330	Notch signaling pathway	3/1108	19/6009	0.708411449108669	0.911171208653728
ko04212	Longevity regulating pathway - worm	11/1108	67/6009	0.713964669905246	0.911171208653728
ko04740	Olfactory transduction	2/1108	13/6009	0.721916154457365	0.911171208653728
ko04744	Phototransduction	2/1108	13/6009	0.721916154457365	0.911171208653728
ko04971	Gastric acid secretion	2/1108	13/6009	0.721916154457365	0.911171208653728
ko00920	Sulfur metabolism	7/1108	44/6009	0.726850172642274	0.911171208653728
ko00130	Ubiquinone and other terpenoid-quinone biosynthesis	8/1108	50/6009	0.727546369718299	0.911171208653728
ko04666	Fc gamma R-mediated phagocytosis	9/1108	56/6009	0.728884506253316	0.911171208653728
ko04921	Oxytocin signaling pathway	11/1108	68/6009	0.732745639135076	0.911171208653728
ko00340	Histidine metabolism	4/1108	26/6009	0.73317208073057	0.911171208653728
ko00604	Glycosphingolipid biosynthesis - ganglio series	1/1108	7/6009	0.76009788012294	0.911171208653728
ko04392	Hippo signaling pathway - multiple species	1/1108	7/6009	0.76009788012294	0.911171208653728
ko00830	Retinol	2/1108	14/6009	0.760246709286158	0.911171208653728

id	Description	Significant	Annotated	Pvalue	Qvalue
	metabolism				
ko03070	Bacterial secretion system	2/1108	14/6009	0.760246709286158	0.911171208653728
ko04071	Sphingolipid signaling pathway	11/1108	70/6009	0.7678175209033	0.911171208653728
ko04916	Melanogenesis	3/1108	21/6009	0.772394273979618	0.911171208653728
ko04720	Long-term potentiation	6/1108	41/6009	0.793663975038574	0.911171208653728
ko00750	Vitamin B6 metabolism	2/1108	15/6009	0.79391031557103	0.911171208653728
ko00909	Sesquiterpenoid and triterpenoid biosynthesis	1/1108	8/6009	0.804385042539432	0.911171208653728
ko04611	Platelet activation	1/1108	8/6009	0.804385042539432	0.911171208653728
ko04977	Vitamin digestion and absorption	1/1108	8/6009	0.804385042539432	0.911171208653728
ko04978	Mineral absorption	1/1108	8/6009	0.804385042539432	0.911171208653728
ko04140	Regulation of autophagy	7/1108	48/6009	0.80781077406744	0.911171208653728
ko04915	Estrogen signaling pathway	6/1108	42/6009	0.812851923406027	0.911171208653728
ko04713	Circadian entrainment	2/1108	16/6009	0.823323247703243	0.911171208653728
ko04972	Pancreatic secretion	3/1108	23/6009	0.824397216612334	0.911171208653728
ko00440	Phosphonate and phosphinate metabolism	1/1108	9/6009	0.840502585093391	0.911171208653728
ko00660	C5-Branched dibasic acid metabolism	1/1108	9/6009	0.840502585093391	0.911171208653728
ko04670	Leukocyte transendothelial migration	1/1108	9/6009	0.840502585093391	0.911171208653728
ko04726	Serotonergic synapse	1/1108	9/6009	0.840502585093391	0.911171208653728
ko04064	NF-kappa B signaling pathway	3/1108	24/6009	0.846356984339265	0.911171208653728
ko04360	Axon guidance	5/1108	38/6009	0.855458247618225	0.911171208653728
ko04014	Ras signaling pathway	6/1108	45/6009	0.862062307103648	0.911171208653728

id	Description	Significant	Annotated	Pvalue	Qvalue
ko04620	Toll-like receptor signaling pathway	3/1108	25/6009	0.865890120180216	0.911171208653728
ko04723	Retrograde endocannabinoid signaling	3/1108	25/6009	0.865890120180216	0.911171208653728
ko04380	Osteoclast differentiation	4/1108	32/6009	0.8670213631285	0.911171208653728
ko00730	Thiamine metabolism	1/1108	10/6009	0.869956441046145	0.911171208653728
ko04923	Regulation of lipolysis in adipocyte	1/1108	10/6009	0.869956441046145	0.911171208653728
ko04974	Protein digestion and absorption	1/1108	10/6009	0.869956441046145	0.911171208653728
ko00790	Folate biosynthesis	2/1108	18/6009	0.87107245751444	0.911171208653728
ko04260	Cardiac muscle contraction	2/1108	18/6009	0.87107245751444	0.911171208653728
ko04013	MAPK signaling pathway - fly	6/1108	46/6009	0.875882363765814	0.911171208653728
ko04724	Glutamatergic synapse	6/1108	46/6009	0.875882363765814	0.911171208653728
ko04115	p53 signaling pathway	4/1108	33/6009	0.882554270876286	0.911171208653728
ko04722	Neurotrophin signaling pathway	7/1108	54/6009	0.89292235011202	0.911171208653728
ko04721	Synaptic vesicle cycle	8/1108	61/6009	0.8976346764305	0.911171208653728
ko00720	Carbon fixation pathways in prokaryotes	3/1108	27/6009	0.898491196625046	0.911171208653728
ko04015	Rap1 signaling pathway	3/1108	27/6009	0.898491196625046	0.911171208653728
ko04510	Focal adhesion	2/1108	20/6009	0.906679079512249	0.911171208653728
ko04621	NOD-like receptor signaling pathway	1/1108	12/6009	0.913560938074992	0.911171208653728
ko04664	Fc epsilon RI signaling pathway	1/1108	12/6009	0.913560938074992	0.911171208653728
ko00620	Pyruvate metabolism	12/1108	89/6009	0.916397606565064	0.911171208653728
ko04110	Cell cycle	19/1108	133/6009	0.917008548473406	0.911171208653728
ko04139	Regulation of	4/1108	36/6009	0.920043936230326	0.911171208653728

id	Description	Significant	Annotated	Pvalue	Qvalue
	mitophagy - yeast				
ko04520	Adherens junction	2/1108	21/6009	0.920814322199894	0.911171208653728
ko04624	Toll and Imd signaling pathway	2/1108	21/6009	0.920814322199894	0.911171208653728
ko04962	Vasopressin-regulated water reabsorption	2/1108	21/6009	0.920814322199894	0.911171208653728
ko00260	Glycine, serine and threonine metabolism	9/1108	71/6009	0.927011445041486	0.911171208653728
ko04011	MAPK signaling pathway - yeast	4/1108	37/6009	0.929919393443923	0.911171208653728
ko00450	Selenocompound metabolism	2/1108	22/6009	0.932914968081848	0.911171208653728
ko04370	VEGF signaling pathway	3/1108	30/6009	0.934123496041419	0.911171208653728
ko04390	Hippo signaling pathway	3/1108	30/6009	0.934123496041419	0.911171208653728
ko04010	MAPK signaling pathway	6/1108	52/6009	0.936485703887477	0.911171208653728
ko03410	Base excision repair	5/1108	45/6009	0.936788343307776	0.911171208653728
ko00230	Purine metabolism	25/1108	175/6009	0.941551190670749	0.911171208653728
ko04924	Renin secretion	3/1108	31/6009	0.943162921348153	0.911171208653728
ko04066	HIF-1 signaling pathway	5/1108	46/6009	0.944215901788504	0.911171208653728
ko04145	Phagosome	11/1108	90/6009	0.958394737023028	0.911171208653728
ko04151	PI3K-Akt signaling pathway	11/1108	90/6009	0.958394737023028	0.911171208653728
ko04142	Lysosome	8/1108	70/6009	0.960108640035898	0.911171208653728
ko04062	Chemokine signaling pathway	1/1108	16/6009	0.96182707120713	0.911171208653728
ko04961	Endocrine and other factor-regulated calcium reabsorption	1/1108	17/6009	0.968884572475694	0.911171208653728
ko04072	Phospholipase D signaling pathway	4/1108	43/6009	0.969268352419851	0.911171208653728
ko04022	cGMP - PKG	5/1108	51/6009	0.970790049783668	0.911171208653728

id	Description	Significant	Annotated	Pvalue	Qvalue
	signaling pathway				
ko04111	Cell cycle - yeast	13/1108	108/6009	0.973473903480087	0.911171208653728
ko00670	One carbon pool by folate	1/1108	18/6009	0.974638226296944	0.911171208653728
ko04650	Natural killer cell mediated cytotoxicity	2/1108	28/6009	0.975873333363053	0.911171208653728
ko04114	Oocyte meiosis	14/1108	116/6009	0.976598272850873	0.911171208653728
ko04113	Meiosis - yeast	7/1108	70/6009	0.982779428908561	0.911171208653728
ko04662	B cell receptor signaling pathway	2/1108	30/6009	0.982990335018785	0.911171208653728
ko04150	mTOR signaling pathway	6/1108	64/6009	0.985764453935617	0.911171208653728
ko00290	Valine, leucine and isoleucine biosynthesis	1/1108	21/6009	0.986269306428565	0.911171208653728
ko04112	Cell cycle - Caulobacter	1/1108	22/6009	0.98880998920698	0.911171208653728
ko00513	Various types of N-glycan biosynthesis	2/1108	33/6009	0.989996152778806	0.911171208653728
ko03060	Protein export	6/1108	67/6009	0.990461074360703	0.911171208653728
ko04530	Tight junction	1/1108	23/6009	0.99088089816951	0.911171208653728
ko04660	T cell receptor signaling pathway	1/1108	23/6009	0.99088089816951	0.911171208653728
ko04141	Protein processing in endoplasmic reticulum	30/1108	238/6009	0.994666430774994	0.911171208653728
ko04914	Progesterone-mediated oocyte maturation	2/1108	37/6009	0.995121551166267	0.911171208653728
ko03430	Mismatch repair	4/1108	56/6009	0.995579089159956	0.911171208653728
ko00510	N-Glycan biosynthesis	2/1108	38/6009	0.995929916238629	0.911171208653728
ko03050	Proteasome	5/1108	66/6009	0.996486437571346	0.911171208653728
ko00190	Oxidative phosphorylation	19/1108	170/6009	0.996736861973201	0.911171208653728
ko03030	DNA replication	5/1108	70/6009	0.998072083525722	0.911171208653728
ko00020	Citrate cycle (TCA cycle)	4/1108	62/6009	0.99828637948519	0.911171208653728
ko03008	Ribosome	8/1108	96/6009	0.998421387151204	0.911171208653728

id	Description	Significant	Annotated	Pvalue	Qvalue
	biogenesis in eukaryotes				
ko03015	mRNA surveillance pathway	9/1108	115/6009	0.999649988543833	0.911171208653728
ko03018	RNA degradation	9/1108	116/6009	0.999695387134438	0.911171208653728
ko00240	Pyrimidine metabolism	12/1108	141/6009	0.99973988855098	0.911171208653728
ko04120	Ubiquitin mediated proteolysis	12/1108	141/6009	0.99973988855098	0.911171208653728
ko03022	Basal transcription factors	2/1108	53/6009	0.999746761257543	0.911171208653728
ko04341	Hedgehog signaling pathway - Fly	17/1108	181/6009	0.9997989610038	0.911171208653728
ko03460	Fanconi anemia pathway	3/1108	66/6009	0.999829495079934	0.911171208653728
ko03020	RNA polymerase	2/1108	56/6009	0.999856223754776	0.911171208653728
ko00970	Aminoacyl-tRNA biosynthesis	2/1108	61/6009	0.999944373293071	0.911171208653728
ko03440	Homologous recombination	3/1108	73/6009	0.999951260768564	0.911171208653728
ko04144	Endocytosis	12/1108	160/6009	0.999979950046619	0.911171208653728
ko04340	Hedgehog signaling pathway	10/1108	152/6009	0.999994376223291	0.911171208653728
ko03420	Nucleotide excision repair	3/1108	87/6009	0.999996220558662	0.911171208653728
ko03013	RNA transport	12/1108	174/6009	0.999997275385684	0.911171208653728
ko03040	Spliceosome	8/1108	212/6009	0.999999999988331	0.911171208653728
ko03010	Ribosome	23/1108	374/6009	0.99999999999965	0.911171208653728

Supplementary Table S4. Detailed comparison of phenylpropanoid pathway and key cold-responsive genes between cold-tolerant (JND815) and cold-sensitive (Jiyu Japonica) rice cultivars during germination under low temperature

Attribute column	Data Column
id	ko00940
Description	Phenylpropanoid biosynthesis
Significant	108/1108
Annotated	254/6009
Pvalue	8.15737278245394e-20
Qvalue	1.88048909406044e-17
Cultivar	JND815 (Cold-tolerant)<br>Jiyu Japonica (Cold-sensitive)
Phenotypic Traits	JND815: Germination rate 89%, MDA 3.2 nmol·g^{-1} FW<br>Jiyu Japonica: Germination rate 43%, MDA 6.8 nmol·g^{-1} FW
Pathway DEG Counts	JND815: Upregulated 98, Cultivar-specific 27<br>Jiyu Japonica: Upregulated 65
Key Gene ID	LOC_Os05g25640<br>LOC_Os06g32980
Gene Family Members	4CL family: LOC_Os10g39170 (log2FC=3.74)<br>PAL family: LOC_Os04g39880 (log2FC=2.83)

Supplemental Table S5. Mapping of Manuscript Labels to Cultivars, Temperature Treatments, and Replicates

Label	Cultivar	Temperature Treatment	Replicate Number
A1	JND815	15℃ (Low-temperature treatment)	Replicate 1
A2	JND815	15℃ (Low-temperature treatment)	Replicate 2
A3	JND815	15℃ (Low-temperature treatment)	Replicate 3
E1	JND815	28℃ (Control treatment)	Replicate 1
E2	JND815	28℃ (Control treatment)	Replicate 2
E3	JND815	28℃ (Control treatment)	Replicate 3
B1	Jiyu Japonica	15℃ (Low-temperature treatment)	Replicate 1
B2	Jiyu Japonica	15℃ (Low-temperature treatment)	Replicate 2
B3	Jiyu Japonica	15℃ (Low-temperature treatment)	Replicate 3
F1	Jiyu Japonica	28℃ (Control treatment)	Replicate 1
F2	Jiyu Japonica	28℃ (Control treatment)	Replicate 2
F3	Jiyu Japonica	28℃ (Control treatment)	Replicate 3

Note: This table clarifies the correspondence between labels (A/E/B/F) used in all figures and tables of the manuscript, and the corresponding cultivars, temperature treatments, and replicates. The numbers "1, 2, 3" in each label represent three biological replicates of the sample.

Supplementary Table S6. Information and Validation Basis of Reference Gene

Reference Gene	Locus ID	GenBank Accession	Amplification Efficiency (E)	R^2	Validation Basis (Stability Evidence)	Reference
Actin1	LOC_Os03g50885	NM_197285.2	98.7%	0.999	Stable expression under abiotic stresses (low temperature, drought, salt) in rice; recommended as internal control for gene expression analysis in japonica rice	Jain et al. (2006) [33]

Notes:(1) Amplification efficiency and R^2 were determined from standard curves in this study; (2) The stability of Actin1 was supported by published studies rather than de novo analysis, as it has been extensively validated for consistency in rice under low-temperature stress.
